# Insertion of a second electrode array—a rare complication of CI reimplantation

**DOI:** 10.1007/s00106-023-01364-0

**Published:** 2023-11-09

**Authors:** M. C. Ketterer, K. Brückerhoff, S. Arndt, R. Beck, A. Aschendorff

**Affiliations:** 1 Department of Otorhinolaryngology, Medical Center—University of Freiburg, Faculty of Medicine, University of Freiburg, Freiburg, Germany; 2 Klinik für Hals- Nasen- Ohrenheilkunde, Universitätsklinikum Freiburg, Medizinische Fakultät, Albert-Ludwigs-Universität Freiburg, Killianstraße 5, 79106 Freiburg, Germany

**Keywords:** Technical upgrade, Reimplantion, Complication, Second electrode array, Scalar position

## Abstract

Due to a technical defect or a medical indication, it may be necessary to explant a cochlear implant. This case report shows that there is the risk of encountering a nonremovable electrode array—as described here from the scala tympani—during cochlear reimplantation. In the present case, insertion of a second electrode array into the free and nonobstructed scala vestibuli was successful. Nonetheless, the indication for reimplantation must be carefully considered, especially in patients with tolerable limitations with little or no loss of speech understanding. Furthermore, surgery should not be performed solely because an implant upgrade is desired.

## History

A 22-year-old patient presented 20 years after the initial implantation of a cochlear implant (22 + 10 Cochlear^TM^, Cochlear Limited, NSW, Sidney, Australia) on the left side with increasing pain in the area of the implant, especially when wearing the speech processor. The hearing impression was the same, but subjective noise was described when wearing the processor, so that the patient used it less often. A revision surgery with dorsocaudal placement had already been performed 10 years earlier due to an implant dislocation toward the mastoid edge. The patient suffered from bilateral congenital sensorineural hearing loss of unknown origin. A syndromic hearing loss could be ruled out. Pregnancy and childbirth proceeded without complications. There was no familial hearing loss and there was also no recurrent otitis, otorrhea, vertigo, and tinnitus.

## Findings

On admission, the auditory canals and eardrums on both sides were free of irritation. The implant site was palpable on the left side more caudally than usual and there was no evidence of infection or hematoma. In the Freiburg language test, monosyllabic understanding was 50% at 65 dB and number understanding was 90% with CI.

## Diagnosis

The indication for revision surgery with an implant replacement was established due to the suspicion of a soft failure with dislocation of the implant and subjectively perceived background noise with unchanged speech understanding.

## Therapy and outcome

**Fig. 1:**
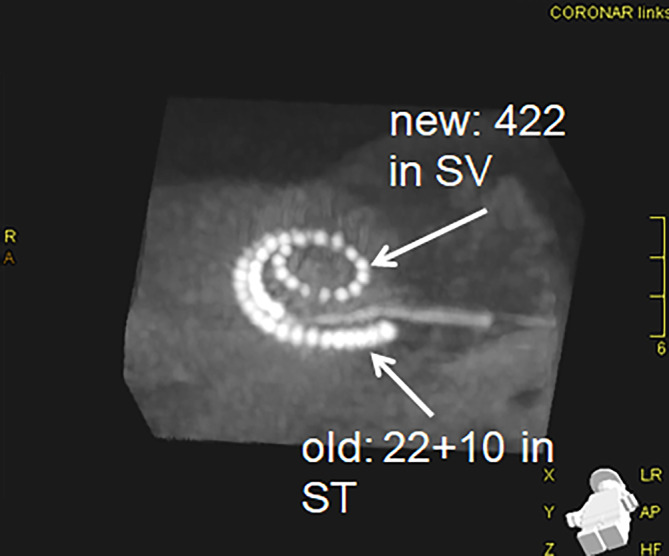
Cone-beam computed tomography image of the petrous bone with original electrode array (*ET*) in the scala tympani (old ET: 22 + 10 Cochlear^TM^) and subsequently inserted electrode array in the scala vestibuli (new ET: 422 Cochlear^TM^)

The electrode array, which was originally inserted into the scala tympani via a cochleostomy, could not be mobilized intraoperatively. The reason for this was reactive osteoneogenesis, which had developed completely around the electrode array, so that it could not be removed even after the cochleostomy had been widened. Initially, the insertion was performed using the Lehnhardt soft surgery technique with preservation of residual hearing. The scala vestibuli was opened, which was free of obliteration and completely open. A test electrode array could be inserted completely. The following insertion of the straight 422 electrode array (422 Cochlear^TM^) into the scala vestibuli was successful and it was decided to leave the non-luxatable electrode array within the scala tympani. This electrode array was cut and the cochleostomy covered with connective tissue. Impedance testing, stapedius reflex measurement, and neural response telemetry (NRT) resulted in regular responses. Postoperative imaging using rotational tomography showed the two electrode carriers in the scala vestibuli and scala tympani (Fig. 1). Speech understanding after reimplantation was at 80% monosyllables and 100% numbers at 65 dB and was stable over 4 years following reimplantation.

## Discussion

Explantation of a cochlear implant may be necessary in the event of a technical defect in the implant or for medical reasons, such as an infection or displacement of the electrode array. A technical defect can be divided into “hard failure” and “soft failure.” A hard failure describes the functional loss of the implant with an objectifiable failure in the integrity test. Soft failure, on the other hand, describes a technically functional implant that does not provide sufficient benefit for the patient or causes other symptoms such as pain or dizziness [12]. The reimplantation of a cochlear implant is a surgical procedure that was first described in 1985 as a possible and successful therapy option in the event of functional failure of an implant [5]. The reimplantation rates for hard and soft failure range from 0.5% to 14.7% in the literature published to date, with the occurrence of this complication described in the literature being higher in children than in adults due to the more frequent impact injuries to the head [12]. Although the overall success rate of reimplantation, measured by speech understanding after revision surgery, is good, these children, in particular, who are in the sensitive phase of speech development, achieve better hearing than with the previous implant [10]. Nevertheless, our own results showed stable speech perception after reimplantation, but no significant improvement despite technical upgrades [3]. Numerous studies have shown that the primary insertion of the electrode array into the scala tympani is preferable and leads to significantly better speech understanding [2, 4]. The insertion quality depends on factors such as the cochlear morphology [7–9] and the surgical learning curve [1]. An implantation of the electrode carrier in the scala vestibuli is possible and also necessary, for example, in the case of otosclerosis or obliteration of the scala tympani. Nevertheless, this can lead to a satisfactory hearing rehabilitation despite partial ossification and scala vestibuli insertion [11]. However, the primary scala vestibuli insertion has not been described as a result of a previous implantation in the scala tympani, but as a given prerequisite before electrode array insertion, for example, due to otosclerosis or infection. The reactive ossification of the scala tympani after electrode array insertion can be caused by the surgical trauma to the cochlea, which causes an inflammatory reaction with subsequent osteoneogenesis [6]. There is also the possibility of metachronous, silent labyrinthitis with ossification as a result. Nevertheless, the subsequent removal and reimplantation of an electrode array is usually possible without complications [12]. In the present case, ossification of the scala tympani occurred with the impossibility of explanting an electrode array, which could only be identified during the surgery. In addition to the neo-ossification, the cause was also the shape of the electrode array (CI22 + 10, Cochlear^TM^, electrode array with ring electrodes), which made extraction impossible.

Even with the currently favored round window insertion with a high percentage of preservation of residual hearing and so-called atraumatic procedure, there is a risk of obliteration and ossification due to silent labyrinthitis, which can lead to unforeseen complications in the case of reimplantation.

The method of implantation in the scala vestibuli is known, but this is the first case report on such a procedure with an already inserted and non-removable electrode array in the scala tympani. The patient achieved good hearing with the new implant, which is comparable to that before reimplantation. Postoperatively, monosyllabic understanding in the Freiburg language test was 80% at 65 dB measured 1 year after reimplantation, which was checked again 4 years after reimplantation and remained stable (Fig. 1). Numerical understanding at 65 dB was 100% 4 years after revision surgery.

This case shows that reimplantation of the cochlea carries the risk of a non-removable electrode array. Although the insertion of a second electrode carrier into an irritation-free scala vestibuli was successful in this case, the indication for reimplantation should be viewed critically and should not be performed because of a possible technical upgrade.

## Declarations

For this article no studies with human participants or animals were performed by any of the authors. All studies mentioned were in accordance with the ethical standards indicated in each case. For images or other information within the manuscript which identify patients, consent was obtained from them and/or their legal guardians.

The supplement containing this article is not sponsored by industry.
